# Evaluation of the Correlation between Gaze Avoidance and Schizophrenia Psychopathology with Deep Learning-based Emotional Recognition

**DOI:** 10.1192/j.eurpsy.2022.803

**Published:** 2022-09-01

**Authors:** H.S. Choi, D.-U. Jung, D.-W. Jeon, S.-J. Kim, J.-J. Moon

**Affiliations:** Inje University Busan Paik Hospital, Psychiatry, Busan, Korea, Republic of

**Keywords:** Psychopathology, eye tracking, schizophrénia, gaze avoidance

## Abstract

**Introduction:**

Direct gaze is the most important mediator of social interaction and communication. Existing studies have evaluated eye movements of patients with schizophrenia by presenting stimuli using photographs or pre-recorded videos, but few directly investigated gaze avoidance in real-world situations.

**Objectives:**

To investigate the correlation between gaze avoidance and psychopathology in patients with schizophrenia through eye movement measurements in real-life interpersonal situations.

**Methods:**

We enrolled 52 clinically stable patients with schizophrenia. Psychopathology was evaluated using the Positive and Negative Syndrome Scale (PANSS), Hamilton Depression Rating Scale, and Hamilton Anxiety Rating Scale. After presenting a visual stimulus, eye movements were measured with Tobii Pro Wearable Glasses 2, and deep learning-based emotional recognition using the residual masking network was used for neutral stimulus verification. Statistical analyses were performed using Pearson’s correlation and regression analyses.

**Results:**

Data of 45 participants with verified stimulus neutrality by deep learning image recognition were used for analysis. The first dwelling time was negatively correlated with the PANSS positive syndrome subscale (p=0.028), general psychopathology subscale (p=0.008), total score (p=0.008), 5-factor positive symptoms (p=0.035), and 5-factor depression/anxiety symptoms (p=0.008). The baseline-area of interest (AOI) pupil diameter change was positively correlated with PANSS 5-factor positive symptom scores (p=0.039). After adjusting for additional variables, the same items had a significant effect on the first dwelling time and baseline-AOI pupil diameter change.

**Conclusions:**

Psychopathology, particularly positive symptoms, was associated with gaze avoidance and pupil diameter in patients with schizophrenia. Evaluating the characteristics of eye movements in patients with schizophrenia will enable better understanding of their symptoms.

**Disclosure:**

No significant relationships.

